# Identification and Expression Analysis of Glucosinolate Biosynthetic Genes and Estimation of Glucosinolate Contents in Edible Organs of *Brassica oleracea* Subspecies

**DOI:** 10.3390/molecules200713089

**Published:** 2015-07-20

**Authors:** Go-Eun Yi, Arif Hasan Khan Robin, Kiwoung Yang, Jong-In Park, Jong-Goo Kang, Tae-Jin Yang, Ill-Sup Nou

**Affiliations:** 1 Department of Horticulture, Sunchon National University, Sunchon 540-950, Korea; E-Mails: yeege91@hanmail.net (G.-E.Y.); gpb21bau@gmail.com (A.H.K.R.); ykw7685@naver.com (K.Y.); jipark@sunchon.ac.kr (J.-I.P.); jgkang@sunchon.ac.kr (J.-G.K.); 2 Department of Plant Science, Seoul National University, Seoul 151-742, Korea; E-Mail: tjyang@snu.ac.kr

**Keywords:** glucosinolates, biosynthetic genes, expression analysis, *Brassica oleracea*, subspecies, edible organs

## Abstract

Glucosinolates are anti-carcinogenic, anti-oxidative biochemical compounds that defend plants from insect and microbial attack. Glucosinolates are abundant in all cruciferous crops, including all vegetable and oilseed *Brassica* species. Here, we studied the expression of glucosinolate biosynthesis genes and determined glucosinolate contents in the edible organs of a total of 12 genotypes of *Brassica oleracea*: three genotypes each from cabbage, kale, kohlrabi and cauliflower subspecies. Among the 81 genes analyzed by RT-PCR, 19 are transcription factor-related, two different sets of 25 genes are involved in aliphatic and indolic biosynthesis pathways and the rest are breakdown-related. The expression of glucosinolate-related genes in the stems of kohlrabi was remarkably different compared to leaves of cabbage and kale and florets of cauliflower as only eight genes out of 81 were expressed in the stem tissues of kohlrabi. In the stem tissue of kohlrabi, only one aliphatic transcription factor-related gene, Bol036286 (*MYB28*) and one indolic transcription factor-related gene, Bol030761 (*MYB51*), were expressed. The results indicated the expression of all genes is not essential for glucosinolate biosynthesis. Using HPLC analysis, a total of 16 different types of glucosinolates were identified in four subspecies, nine of them were aliphatic, four of them were indolic and one was aromatic. Cauliflower florets measured the highest number of 14 glucosinolates. Among the aliphatic glucosinolates, only gluconapin was found in the florets of cauliflower. Glucoiberverin and glucobrassicanapin contents were the highest in the stems of kohlrabi. The indolic methoxyglucobrassicin and aromatic gluconasturtiin accounted for the highest content in the florets of cauliflower. A further detailed investigation and analyses is required to discern the precise roles of each of the genes for aliphatic and indolic glucosinolate biosynthesis in the edible organs.

## 1. Introduction

Glucosinolates, β-thioglucoside-*N*-hydroxysulfates (*cis-N*-hydroximinosulfate esters), are sulfur-enriched, anionic secondary metabolites of plants synthesized from amino acids and sugars. They are synthesized in all vegetables and oilseed plants of the order Brassicales [1]. Upon hydrolysis, these metabolites not only confer characteristic flavors to *Brassica* vegetables [2,3] but also serve to prevent carcinogenesis in animals by regulating the cell cycle and stimulating apoptosis [4]. Hydrolysis by the myrosinase enzyme degrades glucosinolates into different bioactive products, mostly isothiocyanates [5,6,7]. Isothiocyanates such as sulforaphane [8,9] and indole-3-carbinol [10] are strongly anti-carcinogenic, whereas phenethyl isothiocyanate inhibits the transformation of carcinogens from one form to another [11,12]. In addition to their anti-carcinogenic properties in the animals that consume them, glucosinolates are anti-oxidative [13] and help defend against herbivores and microbes [14,15]. Apart from the various benefits of glucosinolates, a few of them, for example progoitrin, are also reported to have adverse effects in animals, with goitrogenic effects (*i.e.*, enlargement of the thyroid) [16], although no evidence of any such effect has been reported in humans from *Brassica* consumption [17]. It is important to understand the genetics of biosynthesis and accumulation of health-promoting glucosinolates in order to increase their content for human and animal health and plant protection.

Plants contain over 200 structurally different glucosinolates, which are generally classified as aliphatic, indolic or aromatic based on their primary precursor amino acids [13,18]. The basic precursors of aliphatic, indolic and aromatic glucosinolates are methionine (or alanine, leucine, isoleucine and valine), tryptophan and phenylalanine (or tyrosine), respectively [13,19]. All three types of glucosinolates are generated by a characteristic biosynthetic pathway that involves elongation of the amino acid side chain by the addition of methylene groups, formation of core structure and subsequent secondary modification of amino acid side chains by oxidation, hydroxylation, methoxylation, sulfation, and glycosylation, *etc.* [20,21,22]. In *Brassica* species, most of the glucosinolates are biosynthesized from methionine. Elongation of the methionine side chain involves methylthioalkylmalate synthase (*MAM*), bile acid:sodium symporter family protein 5 (BASS5) and branched-chain aminotransferase (BCAT) [23,24,25,26]. Formation of the core structure is a five-step process that includes formation of aldoxime by cytochromes P450 of the CYP79 and CYP83 families, oxidation of aldoxime by members of the CYP83 family, formation of thiohydroximic acid followed by C–S cleavage, and formation of desulfoglucosinolate by *S*-glucosyltransferase and glucosinolates by sulfotransferase [27,28]. Subsequent secondary modification involves several gene loci, for example those encoding *GS-OX*, *GS-AOP*, *GS-OH*, *BZO1* and *CYP81F2*. R2R3-Myb transcript factors and other nucleus-localized regulators participate in glucosinolate biosynthesis [18,29,30,31,32,33,34]. Moreover, the sulfate assimilatory pathway, which provides glutathione and 3′-phosphoadenosine 5′-phosphosulfate co-substrates during glucosinolate biosynthesis on desulfo precursor, also involves several other genes [20].

*Brassica oleracea* is an important diversified vegetable species in which it has become clear that glucosinolate biosynthetic and catabolism pathways are different compared to those in *Arabidopsis* and *B. rapa*. *B. oleracea* also shows greater glucosinolate profile diversity than *B. rapa* and *B. napus* [35]. *B. oleracea* and *B. rapa* respectively have 105 and 101 glucosinolate metabolism-related genes, among which 22 genes are related to catabolism [35]. The coding DNA sequences of 84 *B. oleracea* genes related to glucosinolate biosynthesis [35] are available in two independent databases: Bolbase and EnesmblPlants, but expression analysis has been carried out for none of those genes to date. Therefore, a comparative validation of the coding sequences deposited in the two databases is necessary prior to functional analysis. The glucosinolate biosynthesis and catabolism among *Arabidopsis*, *B. rapa* and *B. oleracea* is likely related but also shows variation in the proportion of tandem genes, and the number and functions of genes for *MAM* and 2-oxoglutarate-dependent dioxygenase (*AOP*) [35]. Functions of *MAM* family members for condensation, side chain elongation and chain length production during glucosinolate biosynthesis differs in *Arabidopsis* compared to *B. rapa* and *B. oleracea*. MYB76 transcription factor is present in *Arabidopsis* but *B. oleracea* and *B. rapa* lack in that factor [36]. In addition to 4C glucosinolate, biosynthesis of sinigrin, a 3C glucosinolate, in *B. oleracea* is assumed to be related to high expression of the Bol017070 gene, while its ortholog, Bra013007, remain silenced in *B. rapa* [35]. By contrast, *B. rapa* biosynthesizes more of the 5C glucosinolate glucobrassicanapin due to higher expression of *MAM3* compared to that in *B. oleracea* [35]. *B. oleracea* has only one functional AOP gene (*AOP2*) whereas *Arabidopsis* and *B. rapa* have four and three functional AOP genes, respectively [35].

**Figure 1:**
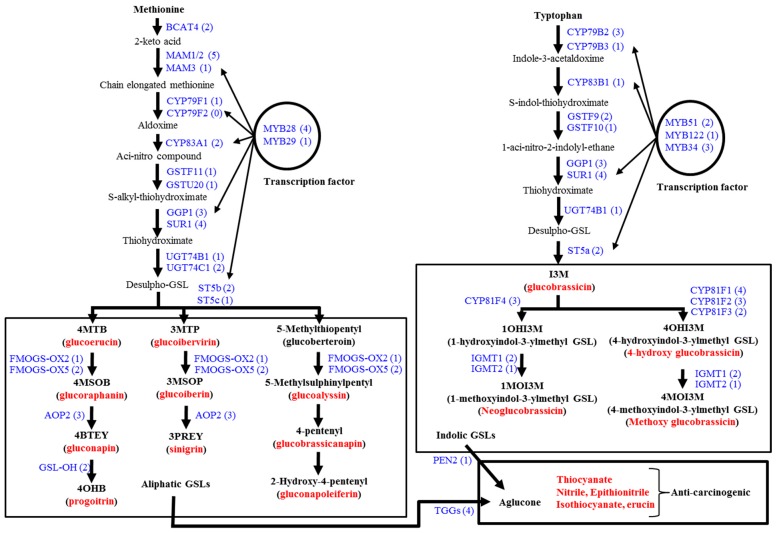
Aliphatic and indolic glucosinolate metabolic pathways along with glucosinolate (GSL)-related genes in *B. oleracea*, after Liu *et al.* [35]. Red bold denotes the GSLs measured in this work by HPLC. Blue bold indicates enzymatic activities for which gene expression was monitored via RT-PCR. Numbers in parentheses are the numbers of genes identified. 4MTB, 4-methylthiobutyl GSL; 4MSOB, 4-methylsulfinylbutyl GSL; 4BTEY, 3-butenyl GSL; 4OHB, 4-hydroxybutyl GSL; 3MTP, 3-methylthiopropyl GSL; 3MSOP: 3-methylsulfinylpropyl GSL; 3PREY: 2-Propenyl GSL; I3M: indolyl-3-methyl GSL.

Under different environmental conditions a complex network of transcription factors from the R2R3-MYB family regulate the glucosinolate biosynthetic pathways [18,29,30,31,32,33,34,37,38,39]. MYB28 and MYB29 are related to biosynthesis of aliphatic glucosinolates [18,33,34,38] while *MYB51*, *MYB122* and *MYB34* are related to indolic glucosinolate biosynthesis (Figure 1) [29,31,32,39]. Transcription factors and/or their stimulators might vary depending on herbivory [12] and sulfur metabolism [40]. Glucosinolate biosynthesis and catabolism differ not only among species [35] but also across developmental stages of tissues and organs during plant development [18,38,39,40,41,42,43,44]. *B. oleracea* is a vegetable-producing species that includes cabbage, broccoli, cauliflower, kale, brussels sprouts and kohlrabi. The edible organs are different in different subspecies; leaf is the edible organ for cabbage (*B. oleracea capitata*) and kale (*B. oleracea acephala*), whereas stem and floret are the edible organs of kohlrabi (*B. oleracea italica*) and cauliflower (*B. oleracea botrytis*), respectively. Currently, little is known about the expression of genes related to glucosinolate biosynthesis at in the edible organs of *B. oleracea* cultivars from qualitative and quantitative perspectives. In this study, we explored the genes related to glucosinolate biosynthesis and studied their expression in the edible organs of *B. oleracea* cultivars. We also measured glucosinolate contents in different edible organs of cabbage, kale, kohlrabi and cauliflower.

## 2. Experimental Section

### 2.1. Plant Materials and Growth Conditions

Seeds of 12 different genotypes from four different groups, three genotypes from each group of *B. oleracea* L., were purchased from Asia Seed Co., Ltd. (Seoul, Korea). The groups were *B. oleracea capitata* (cabbage), *B. oleracea acephala* (curly kale), *B. oleracea italica* (kohlrabi) and *B. oleracea botrytis* (cauliflower) (Table 1). The seedlings were raised in garden soil composed of peat moss, coco peat, perlite, zeolite and vermiculite in a growth chamber. Four-week-old seedlings were transferred to a glasshouse. Plants were grown for four months in the glasshouse before samples were destructively excised from several plants. Sampling sites were the edible organ of the plants (Table 1). The collected samples were snap frozen in liquid nitrogen and freeze-dried and stored at −80 °C for RNA isolation and/or high performance liquid chromatography (HPLC) analysis.

**Table 1:** List of *B. oleracea* subspecies, genotypes and edible organs used to study the expression of glucosinolate-related genes and to estimate content of different glucosinolates.

No.	Common Name	Genotype	Sampling Site/Edible Organ	Scientific Name of the Genotype
1	Cabbage	White cabbage	Leaf	*B. oleracea* L. convar *capitata* (L) Alef. var. alba DC
2	Cabbage	Cabbage	Leaf	*B. oleracea* var. *capitata* alba
3	Cabbage	Sprouting red cabbage	Leaf	*B. oleracea capitata rubra*
4	Kale	Curly kale Halftall	Leaf	*B. oleracea acephala*
5	Kale	Curly kale	Leaf	*B. oleracea* L. convar. *acephala*
6	Kale	Curly kale	Leaf	*B. oleracea* L. convar. *acephala*
7	Kohlrabi	Kohlrabi	Stem	*B. oleracea* var. *italica* Plenck
8	Kohlrabi	Kohlrabi	Stem	*B. oleracea* var. *italica* Plenck
9	Kohlrabi	Kohlrabi	Stem	*B. oleracea* L. convar. *acephala* (DC) Alef. var. *gongyodes*
10	Cauliflower	Cauliflower	Floret	*B. oleracea* L. convar. *botrytis*
11	Cauliflower	Cauliflower	Floret	*B. oleracea* L. convar. *botrytis*
12	Cauliflower	Cauliflower	Floret	*B. oleracea* L. convar. *botrytis*

### 2.2. In Silico Analysis

For *in silico* analysis, the following databases were utilized: *B. rapa* genome database (http://brassicadb.org/brad/glucoGene.php), Bolbase, a comprehensive genomics database for *B. oleracea*, (http://www.ocri-genomics.org/bolbase/index.html) and EnsemblPlants (http://plants.ensembl.org/index.html). Gene symbols and annotated names for glucosinolate-related genes such those for transcription factors and enzymes related to side-chain elongation, core structure formation, secondary modification and co-substrate pathways of *B. rapa* were obtained from the two *B. rapa* databases [42]. *B. oleracea* orthologs along with full-length coding sequence (CDS) and % matching sequence between Bolbase and EnsemblPlants databases were then obtained (Table 2). In cases where Bolbase data were not available, EnsemblPlants data were used.

### 2.3. cDNA Synthesis and Reverse-Transcriptase PCR Analysis

Total RNA of the samples harvested from 12 genotypes of four *B. oleracea* subspecies was extracted using the RNeasy mini kit (Catalogue No. 74106, Qiagen, Valencia, CA, USA). For cDNA synthesis, 5 μg total RNA, 1 μL gene-specific primer, 1 μL annealing buffer and 8 μL RNase were combined in a 0.2 mL thin-walled PCR tube on ice. Gene specific primers were designed using Primer3 website, http://primer3.ut.ee/ (Table 3). PrimeScript-based kit (Takara Bio, Inc., Shiga, Japan) was used for cDNA synthesis. There were two biological replicates for each genotype and gene combination. The RT-PCR experiment was repeated twice for each gene and genotype combination.

**Table 2:** Glucosinolate (GSL) biosynthesis-related genes in *B. oleracea* as compared between Bolbase and EnsemblPlants.

Gene Name	Biosynthesis Pathway	*B. rapa*	*B. oleracea* (Bolbase)	Total CDS of Bolbase	*B. oleracea* (Ensemblplants)	Total CDS of EnsemblPlants	Matching Sequence (%) ***
Transcription factors	Aliphatic and indolic GSLs	Bra012961	Bol007795 (Cun)	558	Bo2g161590.1	1059	554(98.0)
*MYB28*		Bra035929	Bol036286 (C09)	615	Bo9g014610.1	1083	476(93.0)
		Bra029311	Bol017019 (C06)	426	Bo7g098590.1 **	1068	426(99.0)
			Bol036743 (C05)	426	Bo7g098590.1 **	1068	426(98.0)
*MYB29*		Bra009245	Bol008849 (C03)	513	Bo3g004500.1	993	513(94.0)
		Bra005949					
*MYB34*		Bra013000	Bol017062 (C06)	951	Bo7g098110.1	948	951(97.0)
		Bra035954	Bol007760 (Cun)	843	Bo2g161180.1	843	843(97.0)
		Bra029349	Bol036262 (C09)	294	Bo9g014380.1	882	294(100.0)
		Bra029350					
*MYB51*		Bra016553	Bol013207 (C08)	1002	Bo8g067910.1	1002	1002(100.0)
		Bra031035	Bol030761 (C05)	990	Bo5g025570.1	990	990(99.0)
		Bra025666					
*MYB122*		Bra015939	Bol026204 (C07)	981	Bo6g118350.1	1113	981(100.0)
		Bra008131					
*Dof1.1*		Bra030696	Bol023400 (C08)	1005	Bo8g010700.1	1350	915(99.0)
		Bra031588	Bol041144 (C05)	1011	Bo5g008360.1	1011	1011(100.0)
			Bol006511 (C08)	936	Bo8g112940.1	936	936(100.0)
*IQD1*		Bra034081	Bol023096 (C01)	1656	Bo1g144340.1	1368	1260(95.0)
		Bra001299	Bol033935 (Cun)	1437	Bo3g061890.1	1437	1437(99.0)
*TFL2*		Bra023629	Bol021358 (C02)	1146	Bo2g013840.1	1152	1146(94.0)
		Bra013958	Bol000201 (Cun) **	1236	Bo9g159960.1 **	1372	1236(99.0)
		Bra006417	Bol019784 (C09) **	1236	Bo9g159960.1 **	1372	1236(99.0)
			Bol034455 (C03)	999	Bo3g012730.1	1248	906(95.0)
*BCAT-4*	Aliphatic GSLs	Bra022448	Bol018130 (C05)	1083	Bo5g113720.1	1083	1083(99.0)
		Bra001761	Bol026690 (C03)	1083	Bo3g073430.1	825	825(100.0)
*MAM1/2*		Bra029355	Bol017071 (C06)	1236	Bo7g098000.1	1527	1243(81.0)
		Bra013009	Bol020647 (C03)	1518	Bo2g161100.1 **	1518	1518(99.0)
		Bra029356	Bol020646 (C03)	804	Bo2g161100.1 **	1518	760(82.0)
		Bra021947	Bol037823 (C04)	1302	Bo2g102060.1	1494	880(78.0)
		Bra013011	Bol017070 (C06)	1527	Bo7g098000.1	1527	1527(99.0)
*MAM3*		Bra018524	Bol016496 (C02)	759	Bo2g102060.1	1494	759(100.0)
*CYP79F1*		Bra026058	Bol038222 (C05)	1551	Bo5g021810.1	1623	1551(99.0)
*CYP83A1*		Bra032734	Bol040365 (C04)	1506	Bo4g130780.1	1506	1505(99.0)
		Bra016908	Bol005188 (C04)	570	Bo4g191120.1	1509	570(100.0)
*GSTF11*		Bra032010	Bol000843 (Cun)	633	Bo5g150180.1	648	633(98.0)
*GSTU20*		Bra003645	Bol021558 (C07)	654	Bo6g081630.1	654	654(99.0)
*UGT74C1*		Bra021743	Bol006450 (Cun)	1371	Bo4g177530.1	657	647(99.0)
		Bra005641	Bol014127 (C04)	1371	Bo4g049480.1	1372	1371(98.0)
*STb*		Bra015938	Bol026202 (C07)	1035	Bo6g118360.1	1035	1035(100.0)
		Bra015936	Bol026201 (C07)	1035	Bo6g118370.1	1035	1035(100.0)
*STc*		Bra025668	Bol030757 (C05)	1014	Bo5g025610.1	1014	1014(99.0)
*FMOGS-OX2*		Bra027035	Bol010993 (Cun)	1386	Bo9g037180.1	1386	1386(100.0)
*FMOGS-OX5*		Bra016787	Bol029100 (C08)	1347	Bo8g062610.1	1347	1347(99.0)
		Bra026988	Bol031350 (C08)	1380	Bo8g108390.1	1380	1380(100.0)
*AOP2*		Bra034180	Bol045938	?	Bo9g006240.1	1032	
		Bra018521	Bol045939	?	Bo2g102190.1	1104	1105 ****
		Bra000848	Bol045940	?	Bo3g052110.1	948	497 ****
*GS-OH*		Bra022920	Bol033373 (C04) **	243	Bo4g173530.1 **	1077	219(99.0)
		Bra021670	Bol033374 (C04) **	243	Bo4g173530.1 **	1077	219(99.0)
*CYP79B2*	Indolic GSLs	Bra010644	Bol032767 (C03)	1557	Bo3g152800.1	1557	1557(100.0)
		Bra011821	Bol028852 (C01)	1623	Bo1g002970.1	1623	1623(97.0)
		Bra017871	Bol018585 (C06)	1626	Bo7g118840.1	1626	1626(100.0)
*CYP79B3*		Bra030246	Bol031784 (Cun)	1632	Bo4g149550.1	1632	1632(99.0)
*CYP83B1*		Bra034941	Bol033477 (C08)	1473	Bo8g024390.1	1500	1473(100.0)
*GSTF9*		Bra021673	Bol033376 (C04)	648	Bo4g173610.1	648	648(99.0)
		Bra022815	Bol004624 (C03)	648	Bo3g024840.1	648	648(99.0)
*GSTF10*		Bra022816	Bol004625 (C03)	648	Bo3g024850.1	648	648(99.0)
*STa*		Bra008132	Bol039395 (C02)	1014	Bo2g080910.1	1014	1014(100.0)
		Bra015935	Bol026200 (C07)	1017	Bo6g118380.1	1017	1017(100.0)
*CYP81F1*		Bra011762	Bol028913 (C01)	1500	Bo1g003680.1	1437	882(97.0)
		Bra011761	Bol028914 (C01)	1497	Bo1g003710.1	1497	1497(98.0)
			Bol017375 (C07)	369	Bo6g095040.1 **	942	324(99.0)
			Bol017376 (C07)	246	Bo6g095040.1 **	942	232(97.0)
*CYP81F2*		Bra002747	Bol012237 (C09)	933	Bo9g131960.1	1581	933(99.0)
		Bra020459	Bol014239 (C02)	1482	Bo2g032590.1	1482	1482(99.0)
		Bra006830	Bol026044 (C03)	1482	Bo3g019420.1	1482	1482(100.0)
*CYP81F3*		Bra010597	Bol032711 (C03)	1491	Bo3g153480.1	1491	1491(99.0)
		Bra011758	Bol028919 (C01)	1500	Bo1g004740.1	1500	1500(99.0)
*CYP81F4*		Bra010598	Bol032712 (C03)	1506	Bo01007s020.1 **	1506	1506(99.0)
		Bra011759	Bol032714 (C03)	960	Bo01007s020.1 **	1506	807(98.0)
			Bol028918 (C01)	1503	Bo1g004730.1	1506	1503(97.0)
*IGMT1*		Bra012270	Bol007029 (C08)	1119	Bo8g070650.1	1119	1119(100.0)
		Bra012269	Bol020663 (C05)	342	Bo09472s010.1	312	342(100.0)
*IGMT2*		Bra016432	Bol007030 (C08)	1125	Bo8g070660.1	1350	1120(99.0)
GGP1 *	Both aliphatic and indolic GSLs	Bra024068	Bol033672 (C06)	753	Bo7g114570.1	753	753(99.0)
		Bra011201	Bol018073 (C01)	753	Bo1g012070.1	753	753(100.0)
		Bra010283	Bol012989 (C03)	753	Bo3g175530.1	753	753(100.0)
SUR1 *		Bra036490	Bol038767 (C09)	1209	Bo7g003330.1	1371	1209(99.0)
		Bra036703	Bol038764 (C09) **	459	Bo28705s010.1 **	207	207(100.0)
			Bol038765 (C09) **	459	Bo28705s010.1 **	207	207(100.0)
			Bol029775 (Cun)	1008	Bo7g003330.1	1371	991(89.0)
UGT74B1 *		Bra024634	Bol005786 (C05)	1311	Bo5g041080.1	1401	1311(99.0)
*TGG1*	Breakdown aliphatic and indolic GSLs	Bra039825	Bol017328 (C07)	822	Bo6g095780.1	411	373(99.0)
		Bra039824					
		Bra039823					
		Bra016676					
		Bra004012					
*TGG2*		Bra036914	Bol028319 (C08)	1179	Bo8g039420.1	1638	1163(99.0)
			Bol025706 (C03)	663	Bo2g155820.1	714	187(98.0)
*TGG4*							
*TGG5*			Bol031599 (C07)	1326	Bo09266s010.1	163	163(100.0)
*PEN2*		Bra004840	Bol030092 (C04)	1299	Bo4g023800.1	3350	937(99.0)
		Bra004839					

* Participates in biosynthesis of both aliphatic and indolic GSLs; ** These genes have the same CDS sequence in both databases; *** the number of matching base pairs between the sequence in Bol sequence (http://www.ocri-genomics.org/bolbase/) and that in EnsemblPlants (http://plants.ensembl.org/Brassica_oleracea/Info/Index?db=core); **** comparison between *B. rapa* and *B. oleracea* EnsemblPlants sequence.

**Table 3:** Primers used to amplify 81 glucosinolate biosynthetic genes in *B. oleracea* through RT-PCR.

Gene Name	Acc. Number	cDNA Size	Forward	Reverse	Product Size (bp)
**Aliphatic GSL Pathway**					
*BCAT-4*	Bol018130	1083	TACGCGAATGTGAAGTGGGA	CCCCTTCTTATCCTCGACCC	987
	Bol026690	1083	TACGCGAATGTGAAGTGGGA	CACCGTCCACCCCTTCTTAT	996
*MAM1/2*	Bol017071	1236	TGTTGCCCAGTGTGGAAAGG	TGAATGATACAGTTGGCTCCA	970
	Bol020647	1518	GTGACGGCGAACAATCTCC	AGCTTTCCAAGAACAATGCCT	1000
	Bol020646	804	AATGATCCCTACCACCGGTTCAAACA	CTTGCGGCATGTTGATCTCC	650
	Bol037823	1302	CAAGCTTCCCGACACGAATT	CGTCCGCTAAGCTTTCCAAG	975
	Bol017070	1527	GCTCTTACTCCACCGCAGAA	CAACCCCAACATCTTCTGGC	937
*MAM3*	Bol016496	759	CTACCGCCAACACAATCTC	CGTCCGTAATCCTCTTTTTCT	504
*CYP79F1*	Bol038222	1551	GAACCATCGGAGGCAATCAC	AGGTGACGCTCTGGTTTGTA	970
*CYP83A1*	Bol040365	1506	ATGTCAACTTCACGAACCGG	GTTAGGGCCCCACTCTTTCT	953
	Bol005188	570	GGGGTTAACGTTCGTCACTG	GAGCCCAGTCATGACATCCA	442
*GSTF11*	Bol000843	633	TTGGGCAGATAAAAGCAGGT	GCAGCCATTTCCATAAGTTGC	610
*GSTU20*	Bol021558	654	CTGGCCAAGCATGTTCTGTA	TTCCTAAACTCAGCGGCGTA	612
*UGT74C1*	Bol006450	1371	CACACGAACACCCTCAAACC	AGCCTTCACTCTAACCCCAA	989
	Bol014127	1371	CCCTCACGCCAAGATCAAAG	CGGTCTTCACTCTAACCCCA	980
*ST5b*	Bol026202	1035	CGTACCGAACCAAGACAAGA	ACCATGTTCAAGCAAACCTGT	1000
	Bol026201	1035	GGACCAAACCAGGACGAGA	ACCATGTTCAAGCAAACCTGT	999
*ST5c*	Bol030757	1014	TCCAAATCCGAAAACGACGT	GCAAGAAAGCCAGTTCCTCT	995
*FMOGS-OX2*	Bol010993	1386	AGTCTCTCCGAACCAACCTC	AACCACATTCTTCTGCGACC	978
*FMOGS-OX5*	Bol029100	1347	CATAGTCCACTCCAGCGTCT	ACTTGCTCGGCTATCCAGTT	993
	Bol031350	1380	AACCGTAGTCCACTCTAGCG	CTGACCGACGACACCAAGA	970
*GSL-OH*	Bol033373	243	GATTGTGCAAAAGGCTTGT	AGAGCATTAGGATTAGGAGGA	188
	Bol033374	same as Bol033374 CDS			
*AOP2*	Bo9g006240.1		CCAGGAAGTGAGAAGTGGGT	TAGCACCATCACCAGCATCA	517
	Bo2g102190.1		GGAACGTGTCTCCAAAACCC	TAGCACCATCACCAGCATCA	354
	Bo3g052110.1		CCAGGAAGTGAGAAGTGGGT	ACCAACATCCGCACCAGTAT	552
**Indolic GSL pathway**					
*CYP79B2*	Bol032767	1557	GTCAAGTCCTCCTTAGCCGT	CTTGAAGAAGTCTCGCGAGC	219
	Bol028852	1623	ATCACCGTCACATGCCCTAA	CCAGCCCATATCGACTGAGA	991
	Bol018585	1626	CACTCTTACCTCAAACTCTTC	TTTCTTCCGCTCTCTTCT	530
**Indolic GSL pathway**					
*CYP79B3*	Bol031784	1632	CGTCATTCCAGTCACATGTCC	ACGACCAAGTCCGTAACG	1000
*CYP83B1*	Bol033477	1473	GGACCTCAATTTCACCGCTC	TCCACTCCTTTCTGCTCGTT	999
*GSTF9*	Bol033376	648	TGTACGGACCTCACTTTGCT	TCAAGAGTCTCCTTCCAAGCA	613
	Bol004624	648	TGTACGGACCTCACTTTGCT	AAGAGTCTCCTTCCACGCAG	611
*GSTF10*	Bol004625	648	TTGGTGAAGTGCTTGACGTG	CCGGCAATGCGTATTTCTCA	228
*ST5a*	Bol039395	1014	CGGTCTTCACTCTAACCCCA	TCATGTTCAAGCAAGCCAGT	983
	Bol026200	1017	GATCCCAACTCGAGCTCTCA	TCATGTTGAAGCAAGCCAGT	985
*CYP81F1*	Bol028913	1500	GAGACCTCCGCAGTAACCTT	GTCCTCCGTCGGTCTTCTAG	222
	Bol028914	1497	ACTTGATCCTCATCCCTCTCC	CATCGGAGTGAGTTGTGTCAC	483
	Bol017375	369	AAGCAGAGCGGTTCAAGAAG	GCGTGACCATTGTGTTACCA	204
	Bol017376	246	CCGTCTCCTTCAACGGTTCT	CGACGTATTTACCGGTGAGC	170
*CYP81F2*	Bol012237	933	CTACGGAGACCAGGTTCACA	GTCATAATGGGACGCTGATGG	897
	Bol014239	1482	CGTGATCTCTTCTTTGCCCC	TCATCCCATAGCTTCGGGTC	978
	Bol026044	1482	CCAACTCCCTTTCCCATC	TTGCTTTCCCCATCTCTTC	689
*CYP81F3*	Bol032711	1491	TCTCACCCAAAAACCAAC	CTCCCTCCCAATACTTTC	637
	Bol028919	1500	CCTTACTCTCTCCCCATC	CTCCATAATATCTCTTCCCC	478
*CYP81F4*	Bol032712	1506	CGTAGTTGTATCGAACCGCC	TTTCTCCTTCTCCTCCACCG	980
	Bol032714	960	GTTTCAACCTCCCTCCCTCT	CTCTGGGTCTTGTTGTTGCA	818
	Bol028918	1503	CACTCTCTCCCCATTATT	ACCATCTTTATTTCCCCTAC	687
*IGMT1*	Bol007029	1119	GTGTTCCTCTCACCTTCCGA	GTGTTGAGGAAGACGCTGTC	260
	Bol020663	342	AGATGCCATGATCTTGAAACGT	CCAGCAATGATAAGCCTGACA	298
*IGMT2*	Bol007030	1125	TAGGTTTGATGGCCGTGAGA	CGAATTTGCAATGGGTGAAGC	999
**Both aliphatic and indolic GSL pathway**					
*GGP1*	Bol033672	753	TGTTTCTAGCAACTCCTGATTCA	AGTTCTTGCAAATCGTCTCCA	699
	Bol018073	753	TGTTTCTTGCAACTCCTGATTCA	AAATTCTTGCAAATCGTCTCCAA	700
	Bol012989	753	TGTTTCTAGCGACTCTTGATTCA	AAATTCTTGCAAATCGTCTCCAA	700
*SUR1*	Bol038767	1209	TCCGCACCTGTATCGAGG	CTCATCCAGTTCTTCACCCC	1000
	Bol038764	459	CTTCCGTCTATCCTTGCTT	CTGTATCTGTCTTCTTGGT	360
	Bol038765	same as Bol038764 CDS			
**Both aliphatic and indolic GSL pathway**					
*UGT74B1*	Bol029775	1008	TGGTTCCCGCGTTTAAAACT	AGTGGAAGGGTCAGGAGTTA	923
	Bol005786	1311	AATCCTTCAAGCTCAACGGC	TCAAACACCTCACCACCTCA	993
**Breakdown of aliphatic and indolic GSLs**					
*TGG1*	Bol017328	822	TCTTAACGTGTGGGATGGCT	CCTCCTTTGTTCACTCCCCT	210
*TGG2*	Bol028319	1179	AGATGTGCTGGACGAACTCA	CGGCGTAACAGGTAGGATCA	401
	Bol025706	663	CGTTTGGGATGGCTTCAGTC	TTCCTCGGTGAAGTTGGGAA	421
*TGG5*	Bol031599	1326	TGCAGCACATAGAGCACTT	CGGTTCCAGAATCTCCTCC	402
*PEN2*	Bol030092	1299	GCATCATCATCCAACAGCGT	ACGCCTTGATCAGTTCTCCA	207
**Transcription factors for aliphatic and indolic GSL pathways**					
*MYB28*	Bol007795	558	GAGAGGTTCCTTGAGTTGCAAC	GAGAAATCGTAACCCTGATCCA	238
	Bol036286	615	GAAGGTAGCTTGAATGCTAATAC	TATGAGATGCTTTCCGAGGG	414
	Bol017019	426	GTTGCGGCTAAGGTCACTTCT	CAGAAGTAGCGTTGATCTCATGC	223
	Bol036743	426	GGTTTCTTGGGCGCTGCTAC	CCTCGATCATCAACGCTTGTT	328
*MYB29*	Bol008849	513	CGCCCAAGACTTCTGAGTT	TGATATTGCCCATGGAAGCTG	234
*MYB34*	Bol017062	951	TGAGAACACCATGCTGCAAA	ACGAGCTTACAAACTTCTCCA	918
	Bol007760	843	ACCATGTTGCAAAGAAGAAGGA	CCAAACCATCTTCTTCGTTCCA	812
	Bol036262	294	GGTTTCTCCGACAACTGTTCT	ACGAACTCACAAACTTCTCCA	250
*MYB51*	Bol013207	1002	GCTTGTCTCCTACGTCAACC	GTCCTCCTCAAGAAACCTCGA	850
	Bol030761	990	GGACTCCCGAGGAAGATCAG	CCTCGACGTCATTGTTCACA	537
*MYB122*	Bol026204	981	CCTTAGGGCCATCATCAGCT	ACCAGTTGTCAATCCCTTCAA	510
*Dof1.1*	Bol023400	1005	GACGAAACATAGCAGCTCCG	ACCGGGTTGTTCTTCCATCT	227
	Bol041144	1011	TTGGTCACAGCCTACGAACT	TCGTTAGAAGAAGGACCCGA	975
	Bol006511	936	CCAATTGGTCACAGCCTACG	AGAAGGACCCGAGAAATCCG	896
*IQD1*	Bol023096	1656	GGGGTAATTGGAACGACAGC	CTTTCCAACCAGCTCCAACC	202
	Bol033935	1437	CCACAAAACCAGCCGATGAA	AGGATCGTCTTGGTTTGGGT	273
*TFL2*	Bol021358	1146	ACGATGCTGCTGAGAAGTCT	CCTGGTCCCCTTAACTCGTT	199
	Bol000201	1236	CTCTGCGGTTCAGGAGATGGG	CTCCAACACACCAGGATACTC	381
	Bol019784	same as Bol000201 CDS			
	Bol034455	999	CTATCCGTCATAAGCGAGTTC	CGATGTCCAAGTTTGGTGTC	531

EmeraldAmp GT PCR Master Mix Cat, No/ID RR310A (Takara Bio, Inc., Shiga, Japan) was used for PCR mixture preparation. A typical PCR reaction included denaturation at 94 °C for 5 min, followed by 34 cycles at 94 °C for 30 s, 62 °C or 55 °C for 30 s, 72 °C for 1 min, and a final extension at 72 °C for 7 min. The *actin* gene (GenBank accession No. FJ969844) was used as a reference gene as it is expressed consistently in different organs of different species [45,46,47].

### 2.4. Extraction of Desulfo-Glucosinolates and HPLC Analysis

Desulfoglucosinolates from the selected samples were isolated using the HPLC protocol previously used by Choi *et al.* [47] with modifications. Fresh leaf tissue (100 mg) was sampled and snap-frozen in liquid nitrogen and stored at −80 °C freezer. The frozen samples were ground and treated with 1 mL 70% alcohol followed by incubation at 70 °C in a water bath for 10 min and at room temperature for 1 h. The tissue and proteins were precipitated by centrifugation for 8 min at 10,000 *g* at 4 °C and the supernatant was collected for anion-exchange chromatography. The extraction process was repeated twice and the combined supernatant was collected in a 5 mL tube. The combined supernatants represented the crude glucosinolate extracts. The supernatant was mixed with 0.5 mL each 50 mM barium acetate and 50 mM lead acetate. The crude glucosinolates were centrifuged again at 2000 *g* for 10 min and loaded onto a pre-equilibrated column and rinsed three times with 1 mL distilled water and 250 μL aryl sulfatase was added for desulfation. The desulfation process was allowed to continue for 16 h and then the desulfated glucosinolates were eluted with 1 mL distilled water. The eluted glucosinolates were centrifuged at 20,000 *g* for 4 min at 4 °C and passed through a filter to remove any impurities (PTFE, 13 mm, 0.2 μm; Advantec, Pleasanton, CA, USA).

The samples were then used for analysis with an HPLC system (Waters 2695, Waters, Milford, MA, USA) equipped with a C_18_ column (Zorbax Eclipse XBD C_18_, 4.6 mm × 150 mm, Agilent Technologies, Palo Alto, CA, USA). Water and acetonitrile were used as mobile phase solvents. A flow rate of 0.4 mL·min^−1^ was set at 30 °C. The desulfoglucosinolates were detected at 229 nm using a UV-visible detector (PDA 996, Waters) with commercially available sinigrin as a glucosinolate standard for quantification. A sinigrin standard curve was used to quantify the amount of glucosinolates in the samples. Individual glucosinolates were identified after mass spectrometry analysis (HPLC/MS, Agilent 1200 series, Agilent Technologies) using an Electrospray ionization interface operated in positive ion mode.

### 2.5. Statistical Analysis

The HPLC measurements of glucosinolate contents were analyzed via one-way analysis of variance using MINITAB 17 statistical software (Minitab Inc., State College, PA, USA). Pairwise comparisons of means were conducted following Tukey’s procedure for the significant statistical difference.

## 3. Results and Discussion

### 3.1. Genes Related to Glucosinolate Biosynthesis and Breakdown

A total of 84 *B. oleracea* genes orthologous to *B. rapa* genes related to glucosinolate biosynthesis, transcriptional regulation and breakdown were identified (Table 2).These 84 genes were distributed across all nine chromosomes of *B. oleracea* (Supplementary Table S1). Aliphatic biosynthesis, indolic biosynthesis and transcription factor-related genes identified in this study were not clustered in any particular chromosome, but rather were distributed across all nine chromosomes (Supplementary Table S1). The highest numbers of aliphatic and indolic biosynthesis genes were located on chromosomes C4 and C3, respectively (Supplementary Table S1). Nine genes were not able to be assigned to a chromosome and three *AOP2* genes, related to aliphatic glucosinolate biosynthesis, were absent in Bolbase although they were present in EnsemblPlants (Table 2, Supplementary Table S1). Per Bolbase, the highest and the lowest number of glucosinolate genes per chromosome were 14 and four in chromosomes C3 and C2, respectively. There was more than 93% identity in coding sequence (CDS) for 77 genes between Bolbase and EnsemblPlants (Table 2). Three *MAM1/2* genes and one *SUR1* gene, showed 78%–89% identity in the two databases. CDS regions differed in number of nucleotides between the two databases for several genes (Table 2). The aliphatic and indolic glucosinolate pathways involved two sets of 32 genes and seven shared genes including three genes for *GGP1*, four genes for *SUR1* (Bol038764 and Bol038765 have identical CDS) and one gene for *UGT74B1* (Table 2, Figure 1). Twenty genes were transcription related and five genes were related to aglucone biosynthesis through the breakdown of glucosinolates (Figure 1). *MYB28* and *MYB29* are aliphatic transcription factor-related and *MYB51*, *MYB122* and *MYB34* are indolic transcription factor-related genes in *B. oleracea* (Figure 1). The pairs of genes Bol000201 and Bol019784 for *TFL2*, Bol033373 and Bol033374 for *GS-OH*, and Bol038764 and Bol038765 for *SUR1* each shared the same CDS (Table 2).

### 3.2. Glucosinolate-Related Gene Expression in B. oleracea Subspecies

**Figure 2:**
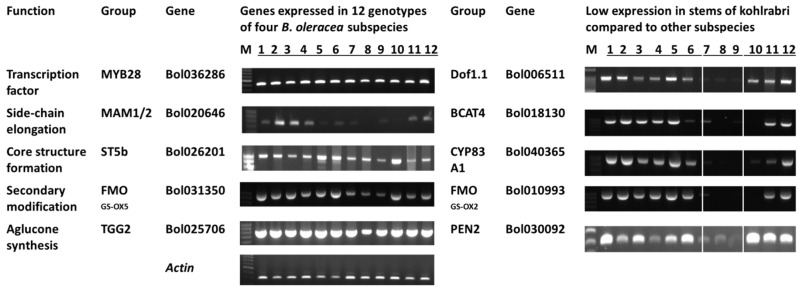
RT-PCR analysis of selected glucosinolate biosynthesis genes reveals differences in expression between stems of kohlrabi and edible organs of three other subspecies of *B. oleracea*. Genotypes 1–3, cabbage; 4–6, kale; 7–9, kohlrabi; 10–12, cauliflower.

Compared to the edible organs of the other three subspecies, the stem samples of kohlrabi had much lower expression of many glucosinolate-related genes (Figure 2, Supplementary Figure S1). However, at least one gene from each of the following categories was expressed in the stems of kohlrabi: transcription factor, core structure formation, secondary modification and aglucone biosynthesis (Figure 2). Expression of side chain elongation-related genes was low in all four subspecies (Figure 2). The majority of other genes were highly expressed only in the leaves of cabbage and kale and in the florets of cauliflower (Figure 2). Only eight genes out of 84 were expressed similarly in the stems of kohlrabi as in leaves of cabbage and kale and floret of cauliflower (Supplementary Figure S1). These genes were *MYB28*, *MYB51*, *IQD1* and *TFL2* of the transcription-factor related set, *AOP2* and *ST5b* aliphatic genes; the *CSTF9* indolic gene and the *TGG2* aglucone biosynthesis-related gene (Supplementary Figure S1). Genotypic differences in gene expression within subspecies for particular genes were also remarkable (Supplementary Figure S1). Some genes yielded notably different product size compared to the expected, for example: *SUR1* (Bol038764, Bol038765), *TGG2* (Bol025706), *TGG5* (Bol031599). The observed product size of Bol038764, Bol025706 and Bol031599 were approximately 850, 800 and 900 bp whereas the expected product sizes from the primers designed based on Bolbase data were 360, 421 and 402 respectively (Supplementary Figure S1). Eight aliphatic pathway genes, three indolic pathway genes, five transcription factor-related genes and one breakdown-related gene were expressed in almost all genotypes of four cultivars (Supplementary Figure S1). These included aliphatic pathway *CYP83A1* (Bol005188), *GSTF11* (Bol000843), *ST5b* (Bol026201, Bol026202), *ST5c* (Bol030757), *FMOGS-OX5* (Bol031350) and *GSL-OH* (Bol033373, Bol033374); indolic pathway *GSTF9* (Bol033376, Bol004624) and *GSTF10* (Bol004625); transcription factor-related *MYB28* (Bol036286), *Dof1.1* (Bol041144), *IQD1* (Bol023096) and *TFL2* (Bol000201, Bol019784) and breakdown-related *TGG2* (Bol025706). Notably, expression levels of *ST5b* (Bol026202) and *GSTF9* (Bol004624) were quite similar across all 12 genotypes (Supplementary Figure S1). Ten genes were expressed only in cabbage, kale and cauliflower but not in the stem of kohlrabi (Supplementary Figure S1). These genes were *CYP83A1* (Bol040365), *ST5a* (Bol026200), *SUR1* (Bol038764, *Bol038765*, Bol029775), *UGT74B1* (Bol005786), *Dof1.1* (Bol023400, Bol006511), *TFL2* (Bol021358), and *PEN2* (Bol030092).

### 3.3. Glucosinolate Analysis in B. oleracea Subspecies

HPLC analysis revealed the presence of 16 different types of glucosinolates in three different edible organs of four different subspecies of *B. oleracea* (Supplementary Table S2, Table 4). Cabbage leaves contained 12 glucosinolates, kale leaves contained 10, kohlrabi stems contained 11 and the cauliflower florets contained 14. Gluconapin, glucoalyssin, gluconapoleiferin and 4-hydroxy glucobrassicin were identified only the florets of cauliflower (Table 4). Glucoerucin was only found in the cabbage leaves, and glucoiberverin was identified in the cabbage leaves and kohlrabi stems (Table 4). The absolute amount of the three aliphatic glucosinolates gluconapin, glucoiberverin and glucobrassicanapin differed significantly or marginally among the edible organs (Table 4). Stems of kohlrabi contained the most glucoiberverin and glucobrassicanapin while the florets of cauliflower contained the highest gluconapin content compared to other edible organs (Table 4). Cauliflower florets recorded the highest content of methoxyglucobrassicin and gluconasturtiin, which are respectively indolic and aromatic glucosinolates (Table 4). Out of 11 aliphatic glucosinolates identified in three types of edible organs in 12 genotypes of four *B. oleracea* subspecies, only gluconapin, glucoalyssin and gluconapoleiferin were expressed in the florets of cauliflower (Table 4, Supplementary Table S3).

**Table 4:** Glucosinolate content (μmol·g^−1^ DW) in edible organs of cabbage, kale, kohlrabi and cauliflower.

Common Name (Edible Organ)	Aliphatic											Indolic				Aromatic
	GER	GRA	GNA	PRO	GIV	GIB	SIN	GAL	GBN	GNL	GRE	GBS	4HGBS	MGBS	NGBS	GST
Cabbage (Leaf)	0.090	1.024	0.000b	0.126	0.004b	1.176	0.065	0.000	0.020ab	0.000	0.851	1.906	0.000	0.078b	0.105	0.141ab
Kale (Leaf)	0.000	0.204	0.000b	0.148	0.000b	0.427	0.201	0.000	0.013ab	0.000	0.074	1.146	0.000	0.126b	0.637	0.119ab
Kohlrabi (Stem)	0.000	0.303	0.000b	0.333	2.115a	1.247	0.601	0.000	0.071a	0.000	0.079	0.434	0.000	0.046b	0.120	0.006b
Cauliflower (Floret)	0.000	0.060	0.053a	0.080	0.000b	0.406	0.073	0.115	0.006b	0.117	0.524	0.252	0.044	1.093a	0.621	1.185a
SE	0.255	0.009	0.040	0.323	0.318	0.133	0.029	0.010	0.029	0.211	0.493	0.008	0.165	0.171	0.187	0.187
*p* (subspecies)	0.44	0.61	0.052	0.10	0.011	0.73	0.50	0.44	0.06	0.44	0.55	0.69	0.07	0.033	0.57	0.056

Different letters indicate significant difference. GER, glucoerucin; GRA, glucoraphanin; GNA, gluconapin; PRO, progoitrin; GIV, glucoiberverin; GIB, glucoiberin; SIN, sinigrin; GAL, glucoalyssin; GBN, glucobrassicanapin; GNL, gluconapoleiferin; GRE, glucoraphenin; GBS, glucobrassicin; 4HGBS; 4-hydroxy glucobrassicin; MGBS, methoxyglucobrassicin; NGBS, neoglucobrassicin; GST, gluconasturtiin.

### 3.4. Discussion

In this study a total of 84 genes related to glucosinolate biosynthesis in *B. oleracea* were compared. Furthermore, the expression of those genes and biosynthesis of glucosinolates in the edible organs were monitored in four *B. oleracea* subspecies. The study revealed a disparity in chromosome position for glucosinolate biosynthesis genes between Bolbase and EnsemblPlants databases (Supplementary Table S1). Moreover, the number of nucleotides in the CDS for several glucosinolate-related genes differs between Bolbase and EnsemblPlants (Table 2). These observations suggest that further investigation and validation of those two databases are required. In a future experimentation cloning and sequencing of the mismatched CDS would be targeted. In the present study, the 84 genes identified and expressed are expected to have high similarity with *Arabidopsis thaliana* and *Brassica rapa*, which have high ancestral synteny [35]. Other than those 84 genes, a recent study revealed that *bHLH04*, *bHLH05*, and *bHLH06*/*MYC2* factors as novel regulators of glucosinolate biosynthesis in Arabidopsis, which belong to basic helix-loop-helix transcription factors and are essential for basal glucosinolate levels and response to jasmonic acid signal pathway; *GTR1* and *GTR2*, which are involved in glucosinolate translocation [48]. Therefore in future investigation these three genes should be also included along with 84 genes reported in Liu *et al.* [35]. A previous study compared 52 glucosinolate biosynthetic genes between *A. thaliana* GLS (AtGS) and the draft *B. rapa* genome using nucleotide BLAST analysis [42]; high nucleotide sequence identity of about 72%–92% for the transcription factor-related genes was noted.

Kim *et al.* [44] studied a total of 17 transcription factor-related genes in *B. rapa* ssp. pekinensis involved in glucosinolate biosynthesis through aliphatic and indolic pathways in leaves, flower, stem and root. Similar to our study, expression of transcription factor-related genes was strikingly different in stem samples compared to leaves and florets [44]. Their relative expression level, compared to the reference gene, in young leaves and flowers was much higher compared to in stem [44], similar to the results of the present study. In *B. rapa*, the highest glucosinolate content was measured in seeds and the lowest in roots and old leaves [44]. The gene Bra035929 (encoding *MYB28*) in *B. rapa* exhibited 16- to 552-fold higher transcript levels in stems compared to seeds, young leaves and roots. Notably, the only *B. oleracea* orthologue of Bra035929, namely Bol036286, was expressed in all three genotypes of stem samples of kohlrabi, along with other edible organs (Figure 2). A *MYB29* gene, Bol08849, an orthologue of Bra005949, which has 11- to 92-fold higher gene expression in stems of *B. rapa* [44], was expressed only in two genotypes of kale and one genotype of kohlrabi (Figure 2). These results are subject of further investigation as those genes were differentially expressed among genotypes within subspecies.

Both transcription factor-related genes and glucosinolate biosynthesis genes showed differences in expression in different plant organs such as seeds, stems, leaves and flowers in previous studies [25,44]. In *A. thaliana*, some important glucosinolate biosynthetic genes, such as *CYP79B2*, *UGT74B1*, *CYP79F1*, *CYP79F2*, *IQD1*, and *Dof1.1*, are expressed only in vascular tissues [19,30,31,49,50,51,52,53]. Desulfoglucosinolate sulfotransferases (BrST) isoforms, involved in core glucosinolate biosynthesis in *B. rapa*, were found to be expressed in mature leaf and root highly compared to other tissues, displaying functional redundancy for differential expression [53]. In our study, the edible organs of kohlrabi (stems) and those of cauliflower (florets) have much different types of structural and vascular tissues compared to the leaves of the other two subspecies analyzed, cabbage and kale, and hence the variation in expression of glucosinolate biosynthesis genes is expected. The fact that only one gene, namely Bol036286, out of five aliphatic transcription factor-related genes was expressed in all 12 genotypes including the stems of kohlrabi suggests that expression of this particular gene is essential in *B. oleracea* to induce desulfo-glucosinolates as a precursor of different aliphatic glucosinolates (Figure 2). Similarly, only one indolic transcription factor-related gene, Bol030761, was expressed in all 12 genotypes, suggesting that the presence of that gene is needed for continuation of the glucosinolate biosynthetic pathway (Supplementary Figure S1). The genes Bol025706 and Bol030092 should be essential for aglucone biosynthesis from the aliphatic and indolic glucosinolate pathways, respectively (Figure 1 and Figure 2). Expression analysis further suggests that in the aliphatic biosynthetic pathway two genes Bol031350 (*FMOGS-OX5*) and Bo9g006240 (*AOP2*) successively carry out glucosinolate transformation in the stems of kohlrabi from the primary glucosinolates glucoerucin and glucoibervirin derived from desulfo-glucosinolates produced by three *ST5* genes (Figure 1, Supplementary Figure S1). Our results thus indicate that: (i) expression of all genes simultaneously is not required for glucosinolate biosynthesis in a particular organ; and (ii) the expression of a single gene or a few genes from each step is required to complete the glucosinolate biosynthesis. In addition, as in the stems of kohlrabi, expression of *MYB28* and contents of aliphatic glucosinolate were detected, but expression of genes related to side-chain elongation were extremely low compared to that in other subspecies, suggesting the involvement of other transcription factors recently reported [48], or there is possibility that glucosinolates were transported.

Glucosinolate concentrations are commonly estimated on a tissue dry weight basis. The variation in glucosinolate concentrations we found in the different edible parts might be related to the fact that leaves, stems and florets have differences in water content. Accordingly, glucosinolate concentration on a tissue fresh weight basis could be different from that on a tissue dry weight basis. Thus, the variation observed in glucosinolate content in our study comparing tissues on a dry weight basis might be explained as a methodological variation.

Velasco *et al.* [54] found that glucosinolate concentration in the floral parts of *B. oleracea acephala* subspecies greatly increases from 300 days of age, but that it decreases rapidly in the leaf samples of the same plants. In this study, we measured glucosinolate concentration only at one time point. Similar to our study, the presence of glucoiberin, sinigrin and glucobrassicin was previously reported in all different subspecies of *B. oleracea* [55,56,57]. Likewise, in this study, other glucosinolates such as glucoraphanin, progoitrin, glucobrassicin, methoxyglucobrassicin, neoglucobrassicin and gluconasturtiin were also expressed in all three types of edible organs, such as in the leaves, stems and florets (Table 4). In *B. oleracea* var. *italica*, the patterns of glucosinolates were found to be mainly controlled genetically and less affected by environmental factors but several agronomic and environmental factors strongly influence the absolute content of various glucosinolates [2,58]. In particular, biosynthesis of aliphatic glucosinolates was found strongly genetically controlled in broccoli whereas that of indolic glucosinolates was controlled by genetic and environmental factors and by their interactions [59,60]. For example, high nitrogen and high sulphur content were found to increase the content of indolic glucobrassicin in cabbage cultivars [61,62].

Glucoraphanin and glucoiberin are the two most desirable glucosinolates from a nutritional perspective, whereas 2-hydroxy-3-butenyl (progoitrin) glucosinolate is undesirable as upon hydrolysis it produces oxazolidine-2-thione, which causes goiters in mammals and other harmful effects [56,63]. Glucoraphanin and glucoiberin were found in all four subspecies (Table 4). In this study, one of the cauliflower genotype measured no progoitrin (Supplementary Table S3). Wang *et al.* [56] found comparatively higher progoitrin in commercial broccoli genotypes compared to inbred lines, 1.77–6.07 μmol·g^−1^ and reported that it contributed around 20% of the total glucosinolates measured in that subspecies. Generally, *B. rapa* is abundant in that undesired glucosinolate [63]. The glucosinolates gluconapin, sinigrin, progoitrin, glucobrassicin and neoglucobrassicin show chemoprotective activity, but produce bitter and pungent isothiocyanates [60], so an excessive content might decrease consumer preference [64]. Cauliflower florets contained all five of these glucosinolates, whereas all but gluconapin were identified in all four subspecies under study (Table 4).

Among the four subspecies, the florets of cauliflower contained the highest number of glucosinolates with the lowest absolute content of progoitrin. This study thus identified that natural variation in glucosinolates and their absolute content exist among the edible organs of different *B. oleracea* subspecies, the results of which might be useful in breeding for glucosinolate contents or in transformation studies.

## 4. Conclusions

In this study, a total of 84 genes related to aliphatic, indolic and aromatic glucosinolate pathways or transcription/breakdown were subjected to RT-PCR-based analysis of expression in the edible organs of four species of *B. oleracea*. Only eight genes were expressed in the stem samples of kohlrabi, whereas majority of those genes were expressed in leaves of cabbage or kale and florets of cauliflower. The results are subject of further investigation as genotypic variation within subspecies is also evident along with subspecies difference. Out of 16 different types of identified glucosinolates, only five differed among the edible organs of four subspecies. Stems of kohlrabi contained the most glucoiberverin and glucobrassicanapin, whereas the florets of cauliflower had the highest contents of glucoraphanin, methoxyglucobrassicin and gluconasturtiin in the four-month-old plants. Overall, cauliflower florets had the highest number of glucosinolates and lacked undesirable progoitrin a genotype-dependent manner.

## Supplementary Information

Supplementary materials can be accessed at: http://www.mdpi.com/1420-3049/20/07/13089/s1.
