# Supplementary material for: Identification and Expression Analysis of Glucosinolate Biosynthetic Genes and Estimation of Glucosinolate Contents in Edible Organs of Brassica oleracea Subspecies
Source: Molecules. 2015 Jul 20;20(7):13089–111. doi: 10.3390/molecules200713089 (PMC6332298; doi:10.3390/molecules200713089)
Supplement: Supplementary file 1 [file molecules-20-13089-s001.pdf]

Supplementary Information

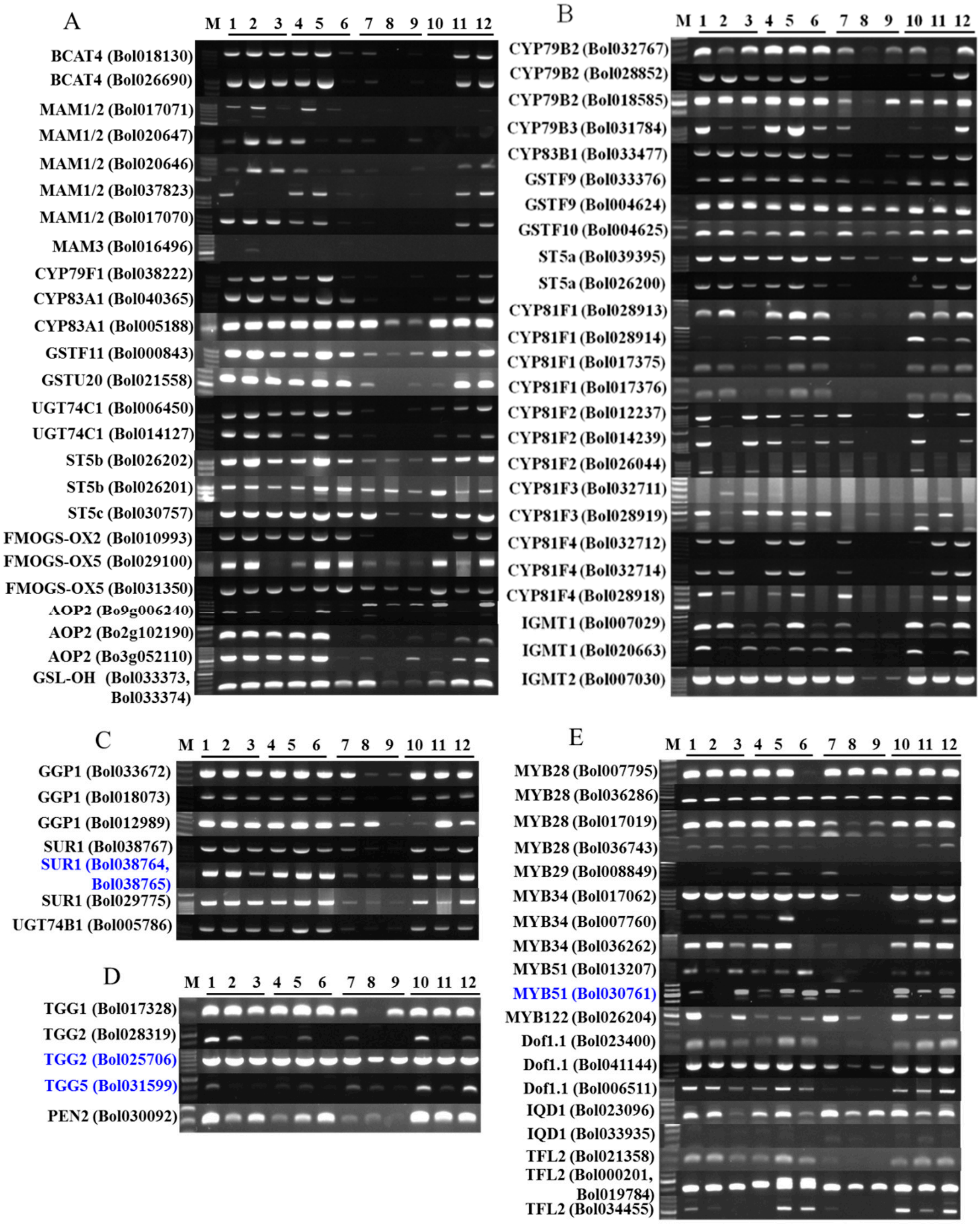

Figure S1. RT-PCR analysis in *B. oleracea* subspecies. Genotypes 1–3, cabbage; 4–6, kale; 7–9, kohlrabi; 10–12, cauliflower. (**A**); Genes for aliphatic GSL enzymes; (**B**); Genes for indolic GSL enzymes; (**C**); Genes for both aliphatic and indolic GSL enzymes; (**D**); Genes related to aglucone GSLs; (**E**); Genes for transcription factors related to GSL biosynthesis. Blue bold indicates that the observed product sizes of the respective gene pairs are different from those predicted based on the primer design.

Table S1. Distribution of 84 glucosinolate biosynthetic genes in nine chromosomes of *Brassica oleracea* based on Bolbase and EnsemblPlants database.

Database	Chr. 1	Chr. 2	Chr. 3	Chr. 4	Chr. 5	Chr. 6	Chr. 7	Chr. 8	Chr. 9	Chr. un	Total
Bolbase	7	4	14	7	9	6	9	9	7	9	81
EnsemblPlants	7	11	11	9	7	9	8	9	7	6	84
Distribution of glucosinolate biosynthesis related genes of different functions based on Bolbase [1]											
Aliphatic		2	4	6	3	2	3	2		3	25
Indolic	5	2	7	1	1	1	3	3	1	1	25
Both	1		1		1	1			3	1	8
Aglucone biosynthesis			1	1			2	1			5
Transcription factor related	1	1	2		4	2	1	3	3	4	21

[1] Three *AOP2* genes are from EnsemblPlants.

Table S2. Glucosinolates identified in the edible organs of *B. oleracea* subspecies by HPLC. The trivial and semi-systematic names and physical properties are shown.

Compound Groups	Trivial Names	Semi-Systematic Names of GSLs	Parental Amino Acid	$[M + H]^+$ (*m*/*z*)	Response Factor	Retention Time
Aliphatic	glucoerucin	4-Methylthiobutyl glucosinolate	Methionine	342	1.00	11.80
Aliphatic	glucoraphanin	4-Methylsulfinybutyl glucosinolate	Methionine	357	1.07	2.473
Aliphatic	gluconapin	3-Butenyl glucosinolate	Methionine	293	1.11	6.145
Aliphatic	progoitrin	(*2R*)-2-Hydroxy-3-butenyl glucosinolate	Methionine	309	1.09	2.100
Aliphatic	glucoiberverin	3-Methylthiopropyl glucosinolate	Methionine	328	1.00	8.400
Aliphatic	glucoiberin	3-Metylsulfinylpropyl glucosinolate	Methionine	344	1.07	1.785
Aliphatic	sinigrin	2-Propenyl glucosinolate	Methionine	279	1.00	2.909
Aliphatic	glucoalyssin	5-Methylsufinylpentyl glucosinolate	Methionine	372	1.07	4.400
Aliphatic	glucobrassicanapin	Pent-4-enyl glucosinolate	Methionine	308	1.15	11.561
Aliphatic	gluconapoleiferin	2-Hydroxy-pent-4-pentenyl glucosinolate	Methionine	324	1.00	4.180
Aliphatic	glucocochlearin	n-Butyl glucosinolate	Methionine	296	1.00	8.000
Aliphatic	glucoraphenin	4-Methylsufinyl-3-butenyl glucosinolate	Methionine	356	1.00	15.768
Indolic	glucobrassicin	3-Indolymethyl glucosinolate	Tryptophan	368	0.29	13.722

Table S2. *Cont.*

Compound Groups	Trivial Names	Semi-Systematic Names of GSLs	Parental Amino Acid	[M + H] + (*m*/*z*)	Response Factor	Retention Time
Indolic	4-hydroxy glucobrassicin	4-Methoxy-3-indolylmethl glucosinolate	Tryptophan	398	0.28	7.199
Indolic	methoxy glucobrassicin	4-Methoxyindol-3-ylmethyl glucosinolate	Tryptophan	399	0.25	16.036
Indolic	neoglucobrassicin	*N*-Methoxy-3-indolylmethyl glucosinolate	Tryptophan	399	0.20	18.593
Aromatic	gluconasturtiin	2-Phenylethyl glucosinolate	Phenyl alanine	343	0.95	14.990

Table S3. Glucosinolate content (μmol g^{-1} DW) in the edible organs of different genotypes of four *B. oleracea* subspecies.

	No.	GER	GRA	GNA	PRO	GIV	GIB	SIN	GAL	GBN	GNL	GRE	GBS	4HGBS	MGBS	NGBS	GST
Cabbage	1	0.270	0.000	0.000	0.119	0.000	0.202	0.042	0.000	0.000	0.000	0.171	0.092	0.000	0.103	0.006	0.095
	2	0.000	0.000	0.000	0.130	0.013	0.100	0.026	0.000	0.012	0.000	0.133	0.060	0.000	0.100	0.002	0.271
	3	0.000	3.071	0.000	0.129	0.000	3.226	0.126	0.000	0.049	0.000	2.249	5.567	0.000	0.029	0.306	0.057
Kale	4	0.000	0.612	0.000	0.293	0.000	0.332	0.557	0.000	0.040	0.000	0.148	0.246	0.000	0.182	0.021	0.268
	5	0.000	0.000	0.000	0.104	0.000	0.044	0.009	0.000	0.000	0.000	0.049	0.032	0.000	0.022	0.007	0.069
	6	0.000	0.000	0.000	0.048	0.000	0.905	0.036	0.000	0.000	0.000	0.025	3.160	0.000	0.175	1.885	0.020
Kohlrabi	7	0.000	0.125	0.000	0.322	3.590	2.967	1.625	0.000	0.076	0.000	0.170	1.066	0.000	0.090	0.093	0.000
	8	0.000	0.784	0.000	0.519	1.834	0.640	0.117	0.000	0.106	0.000	0.059	0.109	0.000	0.023	0.228	0.000
	9	0.000	0.000	0.000	0.157	0.921	0.133	0.060	0.000	0.030	0.000	0.008	0.127	0.000	0.027	0.038	0.018
Cauliflower	10	0.000	0.181	0.078	0.132	0.000	0.714	0.097	0.000	0.019	0.351	1.571	0.552	0.086	0.214	0.378	2.275
	11	0.000	0.000	0.000	0.021	0.000	0.224	0.098	0.000	0.000	0.000	0.000	0.123	0.007	1.325	1.264	0.494
	12	0.000	0.000	0.081	0.087	0.000	0.281	0.024	0.344	0.000	0.000	0.000	0.081	0.038	1.741	0.220	0.786

GER, glucoerucin; GRA, glucoraphanin; GNA, gluconapin; PRO, progoitrin; GIV, glucoiberverin; GIB, glucoiberin; SIN, sinigrin; GAL, glucoalyssin; GBN, glucobrassicanapin; GNL, gluconapoleiferin; GRE, glucoraphenin; GBS, glucobrassicin; 4HGBS, 4-hydroxy glucobrassicin; MGBS, methoxyglucobrassicin; NGBS, neoglucobrassicin; GST, gluconasturtiin.
